# Shifting roles of community health workers in the prevention and management of noncommunicable disease during the COVID-19 pandemic: a scoping review

**DOI:** 10.1093/heapol/czae049

**Published:** 2024-06-24

**Authors:** Tilahun Haregu, Peter Delobelle, Abha Shrestha, Jeemon Panniyammakal, Kavumpurathu Raman Thankappan, Ganeshkumar Parasuraman, Darcelle Schouw, Archana Ramalingam, Ayuba Issaka, Yingting Cao, Naomi Levitt, Brian Oldenburg

**Affiliations:** NCD and Implementation Science Lab, Baker Heart and Diabetes Institute, Melbourne, VIC 3004, Australia; Chronic Disease Initiative for Africa, University of Cape Town, J47/86 Old Main Building, Groote Schuur Hospital Observatory, 7925 Cape Town, South Africa; Department of Public Health, Vrije Universiteit Brussel, Laarbeeklaan 103, 1090 Brussels, Belgium; NCD and Implementation Science Lab, Baker Heart and Diabetes Institute, Melbourne, VIC 3004, Australia; School of Psychology and Public Health, La Trobe University, Plenty Rd, Bundoora, Victoria 3086, Australia; Achutha Menon Centre for Health Science Studies (AMCHSS), Sree Chitra Tirunal Institute of Medical Science and Technology, Trivandrum, India; Public Health, Amrita Institute of Medical Sciences, Kochi, Kerala, India; National Institute of Epidemiology, Chennai, Tamil Nadu, India; Division of Family Medicine and Primary Care, Stellenbosch University, PO Box 241, Cape Town 8000, South Africa; National Institute of Epidemiology, Chennai, Tamil Nadu, India; NCD and Implementation Science Lab, Baker Heart and Diabetes Institute, Melbourne, VIC 3004, Australia; NCD and Implementation Science Lab, Baker Heart and Diabetes Institute, Melbourne, VIC 3004, Australia; Chronic Disease Initiative for Africa, University of Cape Town, J47/86 Old Main Building, Groote Schuur Hospital Observatory, 7925 Cape Town, South Africa; NCD and Implementation Science Lab, Baker Heart and Diabetes Institute, Melbourne, VIC 3004, Australia; School of Psychology and Public Health, La Trobe University, Plenty Rd, Bundoora, Victoria 3086, Australia

**Keywords:** COVID-19, community health workers, noncommunicable diseases, roles

## Abstract

Community Health Workers (CHWs) play a crucial role in the prevention and management of noncommunicable diseases (NCDs). The COVID-19 pandemic triggered the implementation of crisis-driven responses that involved shifts in the roles of CHWs in terms of delivering services for people with NCDs. Strategically aligning these shifts with health systems is crucial to improve NCD service delivery. The aim of this review was to identify and describe COVID-19-triggered shifting roles of CHWs that are promising in terms of NCD service delivery. We searched Ovid Medline, Embase, CINAHL, Web of Science and CABI for Global Health for relevant articles published between 1 January 2020 and 22 February 2022. Studies that were conducted within a COVID-19 context and focused on the shifted roles of CHWs in NCD service delivery were included. We used Preferred Reporting Items for Systematic reviews and Meta-Analyses guidelines to report the findings. A total of 25 articles from 14 countries were included in this review. We identified 12 shifted roles of CHWs in NCD service delivery during COVID-19, which can be categorized in three dimensions: ‘enhanced’ role of CHWs that includes additional tasks such as medication delivery; ‘extended’ roles such as the delivery of NCD services at household level and in remote communities; and ‘enabled’ roles through the use of digital health technologies. Health and digital literacy of people with NCDs, access to internet connectivity for people with NCDs, and the social and organizational context where CHWs work influenced the implementation of the shifted roles of CHWs. In conclusion, the roles of CHWs have shifted during the COVID-19 pandemic to include the delivery of additional NCD services at home and community levels, often supported by digital technologies. Given the importance of the shifting roles in the prevention and management of NCDs, adaptation and integration of these shifted roles into the routine activities of CHWs in the post-COVID period is recommended.

Key messages During the COVID-19 pandemic, the roles of community health workers were:

- enhanced to include additional tasks such as medication delivery
- extended to households and remote communities
- enabled using digital health technologies.

These amplified roles need to be further adapted and integrated into the health systems.

## Introduction

Community health workers (CHWs) have the potential to play a crucial role in the prevention and management of noncommunicable diseases (NCDs) as they are directly involved in providing health services at the community and household levels (Musoke *et al*., 2021). There is strong evidence indicating that CHWs are effective in delivering health education, performing early detection, disease management, adherence support and lifestyle interventions that are essential to managing NCDs at the community level (Jafar *et al*., 2010; Neupane *et al*., 2018; Rawal *et al*., 2020). Existing evidence shows that compared with usual care, involving CHWs in healthcare programmes has produced promising results, including in NCD prevention and management of e.g. tobacco cessation, blood pressure and diabetes control (Neupane *et al*., 2014; Jeet *et al*., 2017). The success of health programmes that involve CHWs is related to careful recruitment of CHWs, detailed hands-on training, empowerment to provide autonomous care, adequacy of medications and supplies, reliable data systems, and fair, performance-based compensation (Heller *et al*., 2019). However, lack of regular supervision and support, inadequate training, lack of clear role definition, involvement in multiple vertical programmes, and insufficient resources have been identified as key barriers to successful CHW-led health interventions (Woldie *et al*., 2018).

Task shifting has been one of the strategies to reduce the workload of the health workforce at higher levels of the healthcare system and improve healthcare delivery at the community level. It also presents a viable option for health system cost savings in low- and middle-income countries (LMICs) (Seidman and Atun, 2017). Task shifting of roles to CHWs has resulted in promising outcomes in HIV/AIDS programmes and maternal and child health programmes (Dawson *et al*., 2014; Ma *et al*., 2016; Limbani *et al*., 2019). Similarly, task-sharing interventions with CHWs for the management of blood pressure in LMICs were found to be effective (Anand *et al*., 2019) and task shifting to frontline CHWs for diabetes care in low-resource settings has also shown promising results (Ogungbe *et al*., 2022). A CHW-led intervention among people with diabetes and/or hypertension in e.g. rural Mexico was associated with improved disease control and adherence to medication (Newman *et al*., 2018). Studies have also shown that CHWs can safely and effectively manage diabetes with the assistance of a mobile application and remote physician supervision (Duffy *et al*., 2020). However, so far, CHW-led interventions for NCDs have not been widely scaled-up in LMICs.

The COVID-19 crisis overloaded health systems with emergency cases and highlighted the important role of CHWs at the community level (Ballard *et al*., 2020). Specifically, CHWs took on adapted roles in the delivery of services for people with NCDs (Peretz *et al*., 2020). These shifted roles have the potential to strengthen the health system for NCD care in the long term. For example, home delivery of medications by CHWs for people with hypertension was found to improve access to medications and medication adherence. The learnings from this intervention can inform the design and implementation of NCD prevention and management policies and programmes

However, the development and implementation of these changes were rapid and they were not institutionalized in local health systems (Elden *et al*., 2023). Strategies for their long-term sustainability and wider scale-up need to be put in place and aligned within health systems, based on evidence to inform adaptation, sustainability and scale-up. The objectives of this scoping review were: (1) to identify and describe the shifting roles of CHWs in the management of NCDs during COVID-19; (2) to highlight factors that affected NCD service delivery by CHWs during the COVID-19 pandemic; and (3) to suggest potential strategies for a better implementation of the shifted roles of CHWs in NCD service delivery. We conducted a scoping review as there were limited studies focusing on changes in the role of CHWs during the COVID-19 pandemic and we aimed to map the literature and identify gaps.

## Methods

### Search strategy

For this scoping review, we searched five scientific databases (Ovid Medline, Embase, CINAHL, Web of Science and CABI Global Health) for relevant articles, using a combination of key terms and synonyms of ‘noncommunicable diseases’, ‘community health workers’ and ‘COVID-19ʹ in our search strategy (Appendix 1, see online supplementary material). An additional search was conducted in a general search engine (Google). The search was limited to English language articles reported after the onset of the COVID-19 pandemic and published between 1 January 2020 and 22 February 2022.

### Study selection

Studies reporting on the role of CHWs in the prevention, treatment and care of NCDs within a COVID-19 context were included. Relevant citations were exported into Endnote software. After de-duplication, we screened the titles and abstracts of the identified articles for eligibility and reviewed full-text papers for inclusion. The key selection criteria were: (1) t he studies should focus on the shifted/modified roles of CHWs; (2) the CHWs’ roles were applied in the prevention, treatment and/or care of common NCDs; and (3) the studies were conducted in the context of COVID-19 restrictions.

We did not restrict the selection by country, study design, study population or publication status. Search, screening and selection of relevant articles were conducted by T.H. and the entire process was rechecked by P.D. Additional articles were identified by reviewing the reference lists of selected articles.

### Definitions

#### CHWs

We used the World Health Organizations definition of CHW: health care providers who live in the community they serve and receive lower levels of formal education and training than professional healthcare workers such as nurses and doctors.

#### Common NCDs

In this study, common NCDs refer to the common NCDs—cardiovascular diseases, diabetes, cancer, chronic lung disease—and diseases collectively considered by authors of the studies as NCDs.

#### Shifted roles

Shifted roles refer to any additional or changed role for CHWs during the COVID-19 pandemic related to NCD prevention and management.

### Data extraction

A structured data extraction template was used to extract the study characteristics and roles of CHWs from the selected articles. We extracted data on study characteristics, including first author, year of publication, title, country, type of intervention involved and the disease/s of focus in the studies. We also extracted qualitative information on the roles of CHWs during the COVID-19 pandemic as reported by the studies, including new roles, challenges and success factors. T.H. reviewed the full text of selected articles and P.D. reviewed and verified the extracted data for completeness and accuracy.

### Synthesis of information

We used thematic synthesis of the qualitative information to describe the major changes identified in the roles of CHWs during COVID-19. First, we highlighted the roles of CHWs in each study. Then, we analysed the types of changes in the role of CHWs during COVID-19 by comparing these with their roles in pre-COVID times. We identified key areas of change from this analysis. We also used thematic synthesis to describe success factors and challenges reported in the implementation of roles of CHWs and their implications for health systems. Based on the findings, we proposed strategies for adaptation, sustainability and scale-up of the shifted roles of CHWs. Results are reported in line with the PRISMA extension for scoping reviews (Tricco *et al*., 2018).

## Results

### Characteristics of included studies

**Figure 1.**
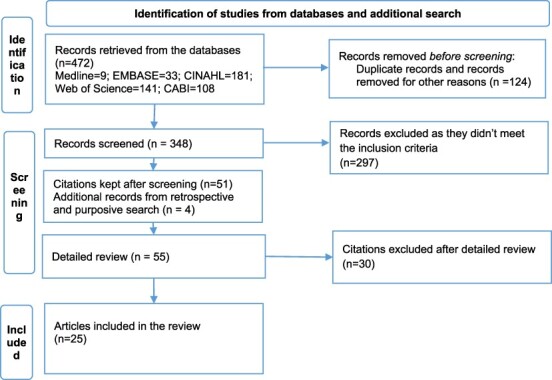
PRISMA flow chart of study identification, screening and selection

A total of 472 articles were retrieved from the databases and 348 articles were retained after deduplication. In all, 51 articles were shortlisted for full-text review after screening of titles and abstracts. Additional search from a general search engine (Google) and screening of reference lists resulted in four more articles that were relevant for this review. Finally, 25 articles met the selection criteria and were included in the review (see Figure 1).

The 25 retrieved papers were from 14 countries. Six of the 25 articles were from the USA, four from South Africa, two from India and two from Brazil. The remaining articles were from Malaysia, Bangladesh, Iran, El Salvador, Japan, Thailand, Philippines, Ethiopia, Indonesia and Jordan. Almost half (12/25) of the studies focused on NCDs in general, while the rest focused on specific NCDs. The study designs included cross-sectional studies, brief/short reports and follow-up studies. The characteristics of the included studies are summarized in Table 1.

**Table 1.** Characteristics of included studies on shifted roles of CHWs in the delivery of NCD services

First author, year	Title	Country	Intervention	Diseases
Brey Z., 2020 (Brey *et al*., 2020)	Home delivery of medication during Coronavirus disease 2019, Cape Town, South Africa: Short report.	South Africa	Home delivery of medication	Chronic diseases
Jamali S., 2020 (Jamali *et al*., 2020)	Personal Experience With COVID-19 and Community Screening of Diabetic Retinopathy in Iran	Iran	Screening of diabetic retinopathy	Diabetes
Kataria I., 2020 (Kataria *et al*., 2020)	Development and evaluation of a digital, community-based intervention to reduce NCDs risk in a low-resource urban setting in Malaysia	Malaysia	Effect change in obesogenic environments and NCD risk	NCD risk (obesity)
Lotta G., 2020 (Lotta *et al*., 2020)	Community health workers reveal COVID-19 disaster in Brazil.	Brazil	Chronic care	Chronic diseases
Mash R., 2020 (Mash *et al*., 2020)	Re-organizing primary health care to respond to the Coronavirus epidemic in Cape Town, South Africa	South Africa	Community-based screening and testing	Chronic conditions
Sampa M.B., 2020 (Sampa *et al*., 2020)	Redesigning Portable Health Clinic Platform as a Remote Healthcare System to Tackle COVID-19 Pandemic Situation in Unreached Communities	Bangladesh	Portable Health clinics in remote areas	NCDs
Soares D.A., 2020 (Soares *et al*., 2020)	COVID-19 tele-screening in SUS users with risk conditions: case report	Brazil	Tele-screening/monitoring of chronic noncommunicable diseases	Chronic diseases
Strand M.A., 2020 (Strand *et al*., 2020)	Community pharmacists’ contributions to disease management during the COVID-19 pandemic	USA	Medication management	Chronic diseases
Ali S.H., 2021 (Ali *et al*., 2021)	Implementation evaluation and fidelity assessment of a diabetes management intervention during the COVID-19 pandemic: findings from the diabetes research, education and action for minorities (DREAM) initiative	USA	Diabetes management	Diabetes
Caperon L., 2021 (Caperon *et al*., 2021)	Identifying opportunities to engage communities with social mobilization activities to tackle NCDs in El Salvador in the context of the global COVID-19 pandemic	El Salvador	Social mobilization	NCDs
Downing K.S., 2021 (Downing *et al*., 2021)	Self-care management 101: strategies for social workers and other frontline responders during the COVID-19 pandemic in rural communities	USA	Self-care management	Chronic health conditions
Lott B.E., 2021 (Lott *et al*., 2021)	Health workers’ perspectives on barriers and facilitators to implementing a new national cervical cancer screening program in Ethiopia	Ethiopia	Referral for screening	Cervical cancer
Merchant R.A., 2021 (Merchant *et al*., 2021)	Community-Based Peer-Led Intervention for Healthy Ageing and Evaluation of the ‘HAPPY’ Program.	Japan	Peer-led dual-exercise program	Mental health
Mistry S.K., 2021 (Mistry *et al*., 2021)	Community Health Workers Can Provide Psychosocial Support to the People During COVID-19 and Beyond in Low- and Middle- Income Countries	LMICs	Psychosocial support	Chronic diseases
Nayawadee K., 2021 (Nayawadee *et al*., 2021)	Community surveillance of COVID-19 by village health volunteers, Thailand	Thailand	Community surveillance	Chronic diseases
Palafox B., 2021 (Palafox *et al*., 2021)	Maintaining population health in low- and middle-income countries during the COVID-19 pandemic: Why we should be investing in Community Health Workers.	Philippines	COVID-19 response	Chronic diseases
Ratnayake R.,2021 (Ratnayake *et al*., 2021)	Adaptation of a community health volunteer strategy for the management of hypertension and diabetes and detection of COVID-19 disease: a programme for Syrian refugees in northern Jordan	Jordan	Disease management	Hypertension and diabetes
Siregar K.N., 2021 (Siregar *et al*., 2021)	Potentials of community-based early detection of cardiovascular disease risk during the COVID-19 pandemic	Indonesia	Self-screening	Cardiovascular diseases
Walker A.F., 2021 (Walker *et al*., 2021)	Using peer power to reduce health disparities: Implementation of a diabetes support coach program in federally qualified health centers	USA	Diabetes coach	Diabetes
Delobelle P.A., 2022 (Delobelle *et al*., 2022)	Non-communicable disease care and management in two sites of the Cape Town Metro during the first wave of COVID-19: A rapid appraisal	South Africa	NCD care and management	NCDs
Harte R., 2022 (Harte *et al*., 2022)	Design of a randomized controlled trial of digital health and community health worker support for diabetes management among low-income patients.	USA	Digital health and CHW support	Diabetes
Mash R.J., 2022 (Mash *et al*., 2022)	Evaluating the implementation of home delivery of medication by community health workers during the COVID-19 pandemic in Cape Town, South Africa: a convergent mixed methods study	South Africa	Medication delivery	Chronic conditions
Vaughan E.M., 2022 (Vaughan *et al*., 2022)	Long-Term Effectiveness of the TIME Intervention to Improve Diabetes Outcomes in Low-Income Settings: a 2 Year Follow-Up	USA	Multidimensional diabetes program	Diabetes
WHO, 2020 (World Health Organization, 2020)	Community drug distribution at doorsteps: Essential health services decentralized to care for hypertensives under the IHCI initiative	India	Home delivery of medications	Hypertension and diabetes
CDC, 2021 (Center for Disease Control, 2021)	Taking Heart: Noncommunicable Disease Detectives Stand Up to COVID-19 in India	India	Remote tracking	Hypertension

### Shifted roles of CHWs

In this review, we identified 12 shifted roles of CHWs in the prevention and management of NCDs during the COVID-19 pandemic. These shifted roles ranged from health promotion and screening to disease management and monitoring (Table 2). During the COVID-19 pandemic, CHWs often provided multiple services when they visited people with NCDs at home, including monitoring and delivery of medications and, in many settings, they served as a critical link between people with NCDs and healthcare providers as follow-up visits to health facilities were disrupted during the pandemic.

**Table 2.** Shifted roles of CHWs in the delivery of NCD services during the COVID-19 pandemic

SN	Shifted roles	Examples
1	Screening/testing and early detection using digital tools	Supporting tele-screening of COVID-19 for chronic disease patients in Brazil (Soares *et al*., 2020) Community screening of diabetic retinopathy in Iran (Jamali *et al*., 2020) Opportunistic screening when delivering medications in South Africa (Mash *et al*., 2020) Facilitators of self-screening using an mHealth application in Indonesia (Siregar *et al*., 2021)
2	Remote monitoring of people with NCDs	Tele-monitoring (monitoring via telephone) of patient progress in Jordan (Ratnayake *et al*., 2021) Remote monitoring using apps (e.g. simple app) in India (Center for Disease Control, 2021) Supporting self-monitoring efforts by patients in the USA (Downing *et al*., 2021)
3	Delivering medications at home for people with NCDs	Community drug distribution at doorsteps in India (World Health Organization, 2020) CHWs delivered medication to the household in Cape Town, South Africa (Delobelle *et al*., 2022)
4	Providing services using portable Health Clinic Platform	Remote healthcare system to tackle COVID-19 pandemic situation in unreached communities in Bangladesh (Sampa *et al*., 2020)
5	Hybrid (face-to-face and virtual) social mobilization	Using a hybrid of virtual and traditional models for social mobilization during COVID-19 in El Salvador (Caperon *et al*., 2021); social resistance in Brazil (Lotta *et al*., 2020)
6	Remotely supporting patients in disease management	Education on self-management, psychosocial support, ensuring sufficient medication, assessing adherence to medication, and screening for complications that required urgent intervention in Jordan (Ratnayake *et al*., 2021)
7	Providing enhanced adherence support to people with NCDs	Assisted patients by counting the remaining medication at their homes Encouraging them to take their medication regularly in South Africa (Mash *et al*., 2022)
8	Providing mental health services at primary health care level	Provide psychosocial support at the community level in a more cost-effective and cost-saving manner as compared to other health professionals (Mistry *et al*., 2021)
9	Using social media network to disseminate information on NCD prevention	Community-based obesity prevention using social media network and mobile application in selected communities in Malaysia (Kataria *et al*., 2020)
10	Technology-enabled referral of patients with complications to health facilities	Referral for cervical cancer screening in Ethiopia (Lott *et al*., 2021) Referral after tracking progress using a mobile application in India (Center for Disease Control, 2021) Referral after telemonitoring and screening for complications in Jordan (Ratnayake *et al*., 2021)
11	Conducting Community surveillance of diseases	Community surveillance of COVID-19 among those with chronic illness such as cardiovascular disease, diabetes or hypertension in Thailand (Nayawadee *et al*., 2021)
12	Facilitating service delivery via telemedicine	CHWs facilitate the use of telemedicine and other innovations that can improve health outcomes of patients with chronic diseases during COVID-19 restrictions (Harte *et al*., 2022)

**Figure 2.**
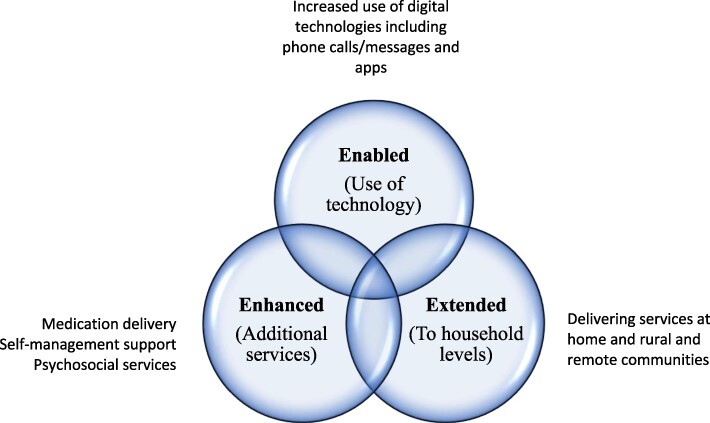
Themes for shifted roles of CHWs in NCD service delivery during the COVID-19 era

Among the shifted roles of CHWs during COVID-19, three interrelated and consistent themes emerged from the literature (Figure 2). Firstly, the roles of CHWs were ‘enhanced’ to include the delivery of additional health services that were not part of their routine practice or were provided less frequently before the COVID-19 pandemic. These included the delivery of hypertension and diabetes medications at home, supporting self-monitoring of people with NCDs at home and providing psychosocial support at home or via phone (Brey *et al*., 2020; World Health Organization, 2020). In pre-COVID times, though CHWs operated at community levels, these NCD services were provided at health facilities during the follow-up visits of people with NCDs to the clinics.

Secondly, the NCD care and treatment services provided by CHWs were further ‘extended’ to the delivery of NCD care to people with NCDs at their homes, including to those living in rural and remote areas (Ngassa Piotie *et al*., 2021; Mash *et al*., 2022). Although CHWs were providing services at community levels and conducted home visits during pre-COVID times, in most settings, their roles in NCD prevention and management were not extended to the follow-up of people with NCDs at their homes. Due to COVID-19 restrictions and the risk of infection that prevented people with NCDs from visiting health facilities, CHWs took an additional role to visit people with diabetes and hypertension at their homes. As part of this, CHWs were involved in the distribution of medications for hypertension and diabetes at home and in conducting opportunistic screening for complications, monitoring of certain markers (e.g. blood pressure and blood glucose), and supporting self-care and adherence to medications (Brey *et al*., 2020; Mash *et al*., 2022).

Thirdly, NCD service delivery by CHWs was ‘enabled’ by the use of digital technologies. Phone calls/messaging and digital apps were used for remote NCD service delivery and monitoring of the health outcomes of people with NCDs. This approach reduced the risk of COVID-19 infection to both CHWs and people with NCDs (Kataria *et al*., 2020; Harte *et al*., 2022). As summarized in Table 2, digital technologies were used in NCD screening/testing, remote monitoring, tracking progress and referral.

### Factors associated with the implementation of the shifted roles of CHWs

For this review, we also extracted and summarized information about factors that were reported to affect the role of CHWs in the provision of services for people with NCDs during the COVID-19 pandemic. We summarized these under the following themes.

#### Factors related to people with NCDs

The general ‘health literacy’ of people with NCDs was associated with improved use of self-monitoring, such as monitoring of blood pressure and blood glucose, and self-management of disease, such as medication adherence and lifestyle modifications. However, the presence of comorbidities among people with NCDs, which makes disease management complex, posed additional challenges to the role of CHWs during the COVID-19 pandemic (World Health Organization, 2020). The digital literacy of people with NCDs was also directly associated with the use of NCD services delivered by CHWs using digital technologies. Lack of correct addresses and out-of-area people with NCDs were also among the challenges in medication delivery at home in South Africa (Delobelle *et al*., 2022; Mash *et al*., 2022).

#### Technology-related factors

There was limited evidence on the technology-related factors that affected the delivery of NCD services by CHWs during the COVID-19 pandemic. However, studies included in this review indicated that the availability of stable ‘internet connectivity’, especially in rural and remote areas, and ‘access to smartphone devices’ for both people with NCDs and CHWs were reported as major challenges to the use of technology-enabled solutions for remote monitoring of people with NCDs (Kataria *et al*., 2020; Center for Disease Control, 2021; Harte *et al*., 2022). To address these challenges, digital applications with offline capabilities and the use of mobile text messages were found to be useful.

#### CHW-related factors

CHW-related challenges were mainly related to the low value and limited recognition given to CHWs in the health system pre-COVID, as well as lack of formalized training, stable employment and remuneration (Stansert Katzen *et al*., 2022). In this review, we also found that a range of other factors influenced CHWs during COVID. These included inadequate ‘training’ for CHWs for expanded roles (e.g. on handling of temperature-sensitive medication), and ‘anxiety and fears’ among CHWs about visiting homes of people with NCDs because of the risk of COVID-19 infection. CHWs’ limited ‘access to disinfection facilities’ (including sanitizers and personal protective equipment) in the communities was also among the challenges. In many settings, there were limited ‘numbers of CHWs’ to reach all people with NCDs in the community. Besides, ‘information overload’ (too much information to CHWs via emails, text messages, etc.) and increased ‘overall workload’ were the main challenges associated with the shifted roles of CHWs during the COVID-19 pandemic (Delobelle *et al*., 2022; Mash *et al*., 2022). Moreover, CHWs’ involvement in the home delivery of medications and monitoring people with NCDs has exposed them to queries from people with NCDs about medication side-effects and interpretation of blood pressure and blood glucose monitoring results. However, close ‘collaborations’ between CHWs and their communities and between CHWs and the health system facilitated the roles of CHWs during the COVID-19 pandemic.

#### Other health system-related factors

**Figure 3.**
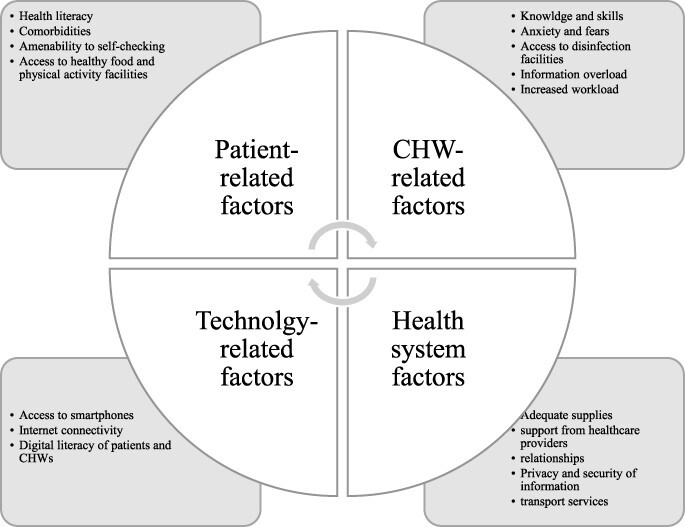
Success and challenge factors in shifted roles of CWHs in NCD service delivery during COVID-19 pandemic

Other health system challenges experienced by CHWs were exacerbated during the COVID-19 pandemic period. Along with the high priority given to the COVID-19 response, the lack of preparedness of the health system for an NCD response and the limited resources available to address NCDs during the crisis period were the underlying factors that affected the delivery of NCD services during the pandemic. For example, home delivery of medications by CHWs, lack of medications at health facilities, and lack of timely ‘support from other healthcare workers’ were found to be among the most challenging factors that affected the role of CHWs in supporting people with NCDs. ‘Inadvertent disclosure’ of private information about people with NCDs to family members during the delivery of medications and ‘transport challenges’ associated with medication distribution were encountered in South Africa (Mash *et al*., 2022). Figure 3 summarizes the details of these factors.

## Discussion

In this review, we found that the roles of CHWs were shifted during COVID-19 restrictions. As people with NCDs could not visit health facilities due to COVID-19 restrictions, CHWs’ role as the primary link between the healthcare providers and people with NCDs, to deliver NCD services at the community level and homes, became very important. As a result, the roles of CHWs were enhanced with additional responsibility to monitor people with NCDs and deliver medications, extended to deliver NCD services at households, and enabled by the digital technology (in many instances) for remote screening/monitoring and support of people with NCDs in self-care and self-monitoring. In contrast to pre-COVID-19 times (Hartzler *et al*., 2018; Javanparast *et al*., 2018), the roles of CHWs included additional activities, and existing activities were also expanded. However, factors such as health literacy, access to internet connectivity and the limited number of CHWs affected the implementation of these shifted roles.

Though the geographical yield of the reviewed studies may to some extent reflect the region-specific search terms, the high representation of high-income countries in this review may reflect the disproportionately low health research evidence from LMICs during the COVID-19 pandemic. Other studies have reported a low representation of research evidence from LMICs (Yegros-Yegros *et al*., 2020; Dimitris *et al*., 2021). Even though CHWs are one of the main health service providers at community levels in LMICs, our review indicated that the representation of LMICs in this review was similar during the COVID-19 pandemic. However, the lack of evidence in this review refers to the included studies only. Nevertheless, future research focusing on the role of CHWs in NCD prevention and control needs to give more attention to LMICs.

Given the increasing incidence of NCDs and recent changes in the health systems of many countries, including the increasing use of digital health solutions by CHWs, it is recommended to explore further adaptations of these shifted roles for better integration into the health system to ensure future sustainability and wider scale-up. The findings of this review may therefore have important implications for policy, practice and research in the following key areas.

### Strengthening collaboration between CHWs and facility-based healthcare providers

Strengthening the collaboration between CHWs and facility-based healthcare provides can provide a strong institutional anchor for the roles of CHWs (Sarriot *et al*., 2021). Given the structural and institutional challenges faced by CHWs, a stronger collaboration between CHWs and other healthcare providers would improve the delivery of NCD services for people with NCDs. We found that the implementation of home delivery of medications during the COVID-19 pandemic required frequent interactions between facility-based health workers, CHWs and other stakeholders as these teams had to work closely and communicate more frequently in the planning, preparation and delivery of medication as well as in the monitoring of medication use by people with NCDs. CHWs served as a critical link between people with NCDs and facility-based health workers during the COVID-19 pandemic. As a result, there has been an increasing recognition of the importance of this collaboration (Salve *et al*., 2023). Building on the lessons learned during COVID-19 restrictions, advancing the increased interaction between CHWs and healthcare providers to a stronger collaboration may improve the delivery of NCD services at community and household levels.

### Training CHWs on the expanded roles

CHWs played a critical role in the response to the unprecedented COVID-19 pandemic. The roles of CHWs shifted substantially during the pandemic (Bhaumik *et al*., 2020). Even though, in some settings, CHWs were exposed to increased risk of infection, these shifts provided CHWs with the opportunity to acquire important new skills and experience in health service delivery for people with NCDs. Recognition of the new skills of CHWs in the delivery of NCD services by the health system is important for the sustainability of their shifted roles (Astale *et al*., 2023). Besides, more tailored training on NCD management could address the remaining knowledge and skills gaps of CHWs in the delivery of NCD services at community and household levels and improve the quality of NCD services delivered to people with NCDs. As the COVID-19 situation transitions to normalcy, a more consolidated CHW training in delivering NCD services at the community level will be crucial (LeBan *et al*., 2021). Perhaps CHWs could play an essential role in the delivery of comprehensive services for infectious diseases, NCDs, and maternal and child health (Akselrod *et al*., 2023; Mfinanga, 2023).

### Increasing the value of home visits

The lessons learned from the shifted roles of CHWs in the delivery of NCD services during the COVID-19 pandemic emphasize the importance of home visits in the provision of NCD care for people with NCDs. In this regard, health systems could strategically increase the value of home visits by defining NCD services that can be delivered by CHWs during home visits (Rahman *et al*., 2021; Ballard *et al*., 2022). Our review has shown that CHWs have the potential to deliver important NCD services, such as tracing people with NCDs who are not retained in care and referring people with NCDs who need clinical care to health facilities. If resources constraints and operational challenges are addressed, CHWs could visit more people with NCDs in their catchment area more frequently, and by combining these visits with service delivery, they could increase the reach and coverage of essential NCD services. Our findings suggest the need to build upon the experience of home delivery of NCD services during COVID-19 restrictions to improve community-level care of people with NCDs. This is in line with the recommendations of the National Noncommunicable Disease and Injury Poverty Commission’s recommendation on using the learnings from the role of CHWs in maternal and child health services to improve NCD prevention and management (Bukhman *et al*., 2020).

### Promoting the hybrid model

Based on the lessons learned from NCD service delivery during the COVID-19 pandemic, a hybrid model, a combination of face-to-face NCD service delivery by CHWs (e.g. medication delivery at home) and remote monitoring and support by healthcare providers, could improve the delivery of NCD services at community and household levels. This model could be a better option in areas with good internet connectivity and better access to smartphone devices. However, to address the disparities that are associated with access to digital health solutions, delivery of NCD services at the community level by CHWs could be considered a viable option to improve access and quality of care for people with NCDs in LMICs (Ganjali *et al*., 2022). Such an approach would need to consider the wider social, structural, institutional and organizational contexts under which CHWs work (Haregu *et al*., 2023). For example, CHWs vary widely in their training and experience; some are volunteers, while others are salaried workers. They are often expected to provide many other services, including maternal and child health and infectious disease services. Additionally, in some settings, CHWs have community-level health posts, while in others, they do not.

### Towards health equity

Given the unequal and unfair distribution of the NCD burden in resource-constrained settings and among socially disadvantaged populations in high-income settings, CHWs can play a crucial role in the prevention and management of NCDs. However, CHWs generally have low status and recognition in the primary healthcare system in these settings. Enhancing their status and increasing their recognition would significantly contribute to reducing the NCD burden in these populations. This will also ensure equity in the delivery of NCD services.

There are some limitations associated with this review. First, it covered a very short period of publication and may not cover studies that were still in progress during the review period. Second, there was significant heterogeneity among the studies included in this review and they did not provide a detailed account of the roles of the CHWs and the challenges they faced. Thus, the synthesis of results focused on higher-level themes. Further studies are needed to expand and deepen the evidence on these themes. Third, CHWs were described by various terms in different countries and languages. Although we used key descriptors, our list of search terms for CHWs was not exhaustive. A more diverse search would help to capture the literature that can explain the structural, organizational, institutional and cultural issues that affect the roles of CHWs in various health systems Fourth, grey literature was not included in this review and high-income countries were overrepresented. This may affect the representativeness of the shifted roles of CHW in LMICs. Finally, during the COVID-19 pandemic, local health system dynamics have been rapidly changing and the studies included in this review may not reflect this reality at the local level.

## Conclusions

The COVID-19 pandemic has resulted in a shift in the roles of CHWs in the delivery critically needed NCD services at community and household levels at times of COVID-19 restrictions. These roles are also useful for post-pandemic health system resilience and preparedness efforts. As health systems deal with global factors such as pandemics, the health impacts of climate change, existing and emerging health burdens, and other health systems challenges, CHWs can play a critical role. Adaptation of the CHWs’ shifted roles in the delivery of essential NCD services, along with strengthening of the capacity of CHWs with additional knowledge and skills relevant to NCD care and increasing the value of home visits by CHWs, may improve health outcomes of people with NCDs.

## Supplementary Material

czae049_Supp

## Reflexivity statement

The author group includes five female and seven male researchers, four senior researchers and eight early and mid-career researchers. They are from Australia India and South Africa.
