# Supplementary material for: Shifting roles of community health workers in the prevention and management of noncommunicable disease during the COVID-19 pandemic: a scoping review
Source: Health Policy Plan. 2024 Jun 24;39(7):771–81. doi: 10.1093/heapol/czae049 (PMC11308610; doi:10.1093/heapol/czae049)
Supplement: czae049_Supp [file czae049_supp.zip › suppl_data/Annex 1.docx]

**Annex 1: Search strategy**

**NCDs/chronic diseases terms**

1. (non-communicable diseases or ncd or noncommunicable diseases)
2. Chronic disease
3. (Diabetes type 2 or diabetes mellitus type 2 or diabetes 2)
4. (Hypertension or high blood pressure)
5. 1 OR 2 OR 3 OR 4

**COVID-19 terms**

1. Covid-19
2. Coronavirus
3. 2019-ncov
4. Sars-cov-2
5. Cov-19
6. 6 0R 7 OR 8 OR 9 OR 10

**Community health workers terms**

1. Community health worker
2. Community health volunteer
3. Accredited social health activist
4. ASHA
5. Women health volunteers
6. Promotora de salud
7. Health promoter
8. 12 OR 13 OR 14 OR 15 OR 16 OR 17 OR 18

**Combining the terms**

1. 5 AND 11 AND 19
